# Supplementary figures and images for: Genetic and structural study of DNA-directed RNA polymerase II of Trypanosoma brucei, towards the designing of novel antiparasitic agents
Source: PeerJ. 2017 Mar 1;5:e3061. doi: 10.7717/peerj.3061 (PMC5335688; doi:10.7717/peerj.3061)

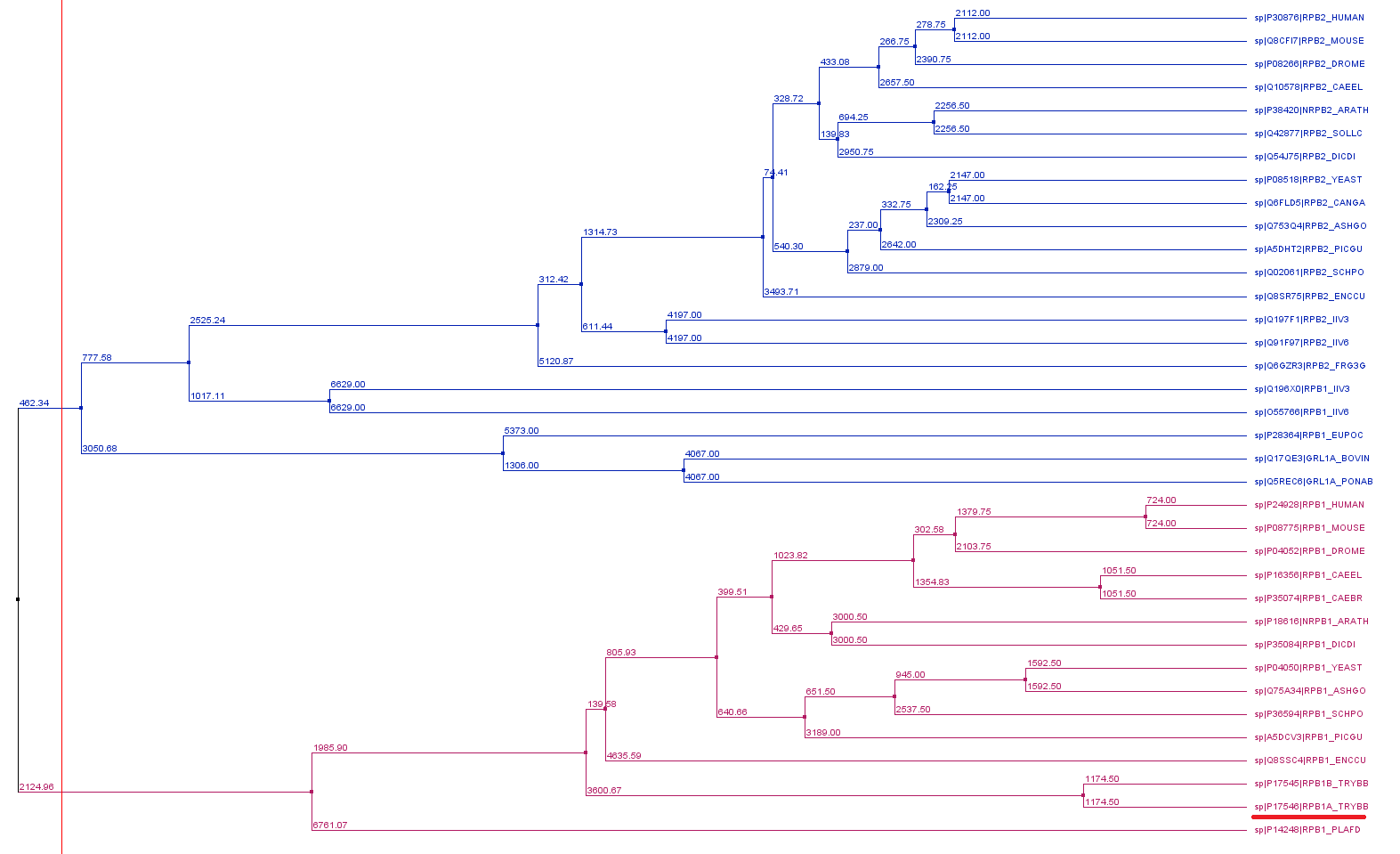

Supplement: Figure S1 — The tree was generated using the DdRpII family dataset (36 foul length protein sequences samples) and the Jalview software. Tree was constructed using the average distance statistical method with PAM 250. In the tree representation there are clearly shown the two RNA polymerases II subunits RPB1 and RPB2 as two main monophyletic sub-trees. Trypanosoma brucei DdRpII RPB1 protein sequence was correctly classified in the monophyletic sub-tree of the RPB1 group. [file peerj-05-3061-s001.png]

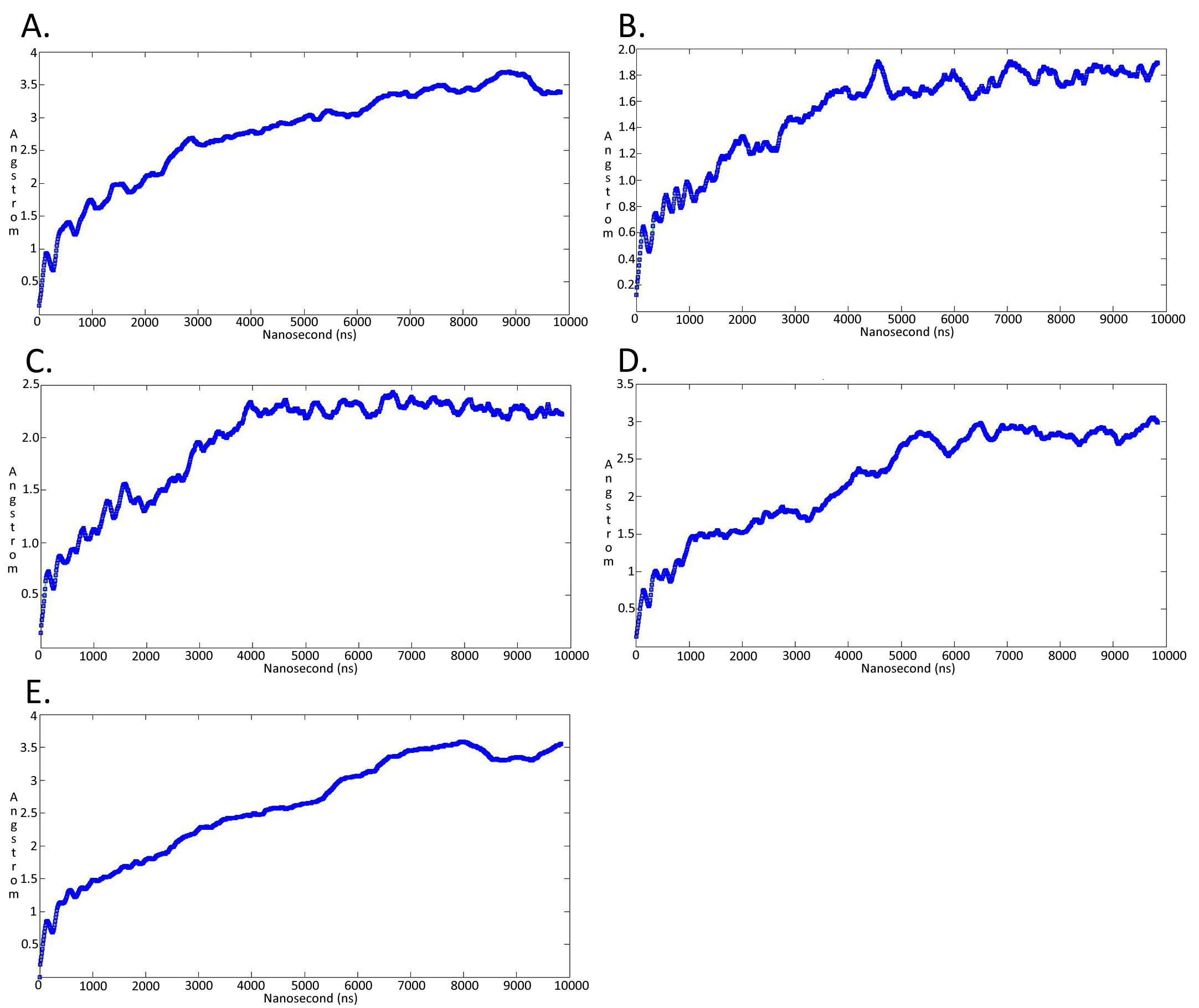

Supplement: Figure S4 — The energy (Kcal/mol) vs time (ns) plot of the 100ns simulation trajectory of the TBB DdRpII RPBI model A. Sub-domain regions of the Trypanosoma brucei brucei DdRPII RPB1 have been separated according to conventions of Fig. S3. (A) Domain A RMSD. (B) Domain B RMSD. (C) Domain C RMSD. (D) Domain D RMSD. (E) Domain E RMSD. [file peerj-05-3061-s004.png]

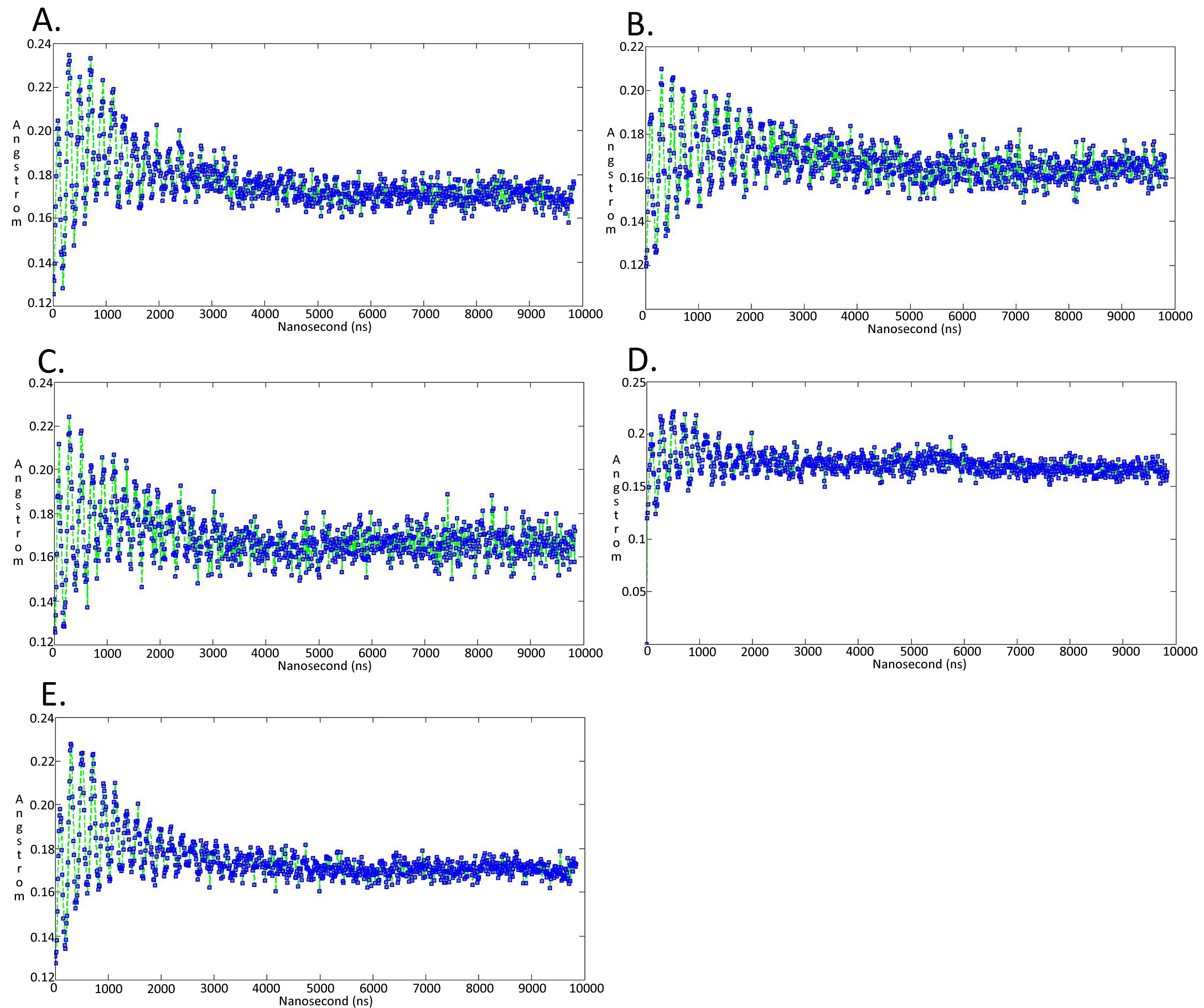

Supplement: Figure S5 — Sub-domain regions of the Trypanosoma brucei brucei DdRPII RPB1 have been separated according to conventions of Fig. S3. (A) Domain A RMSF. (B) Domain B RMSF. (C) Domain C RMSF. (D) Domain D RMSF. (E) Domain E RMSF. [file peerj-05-3061-s005.png]

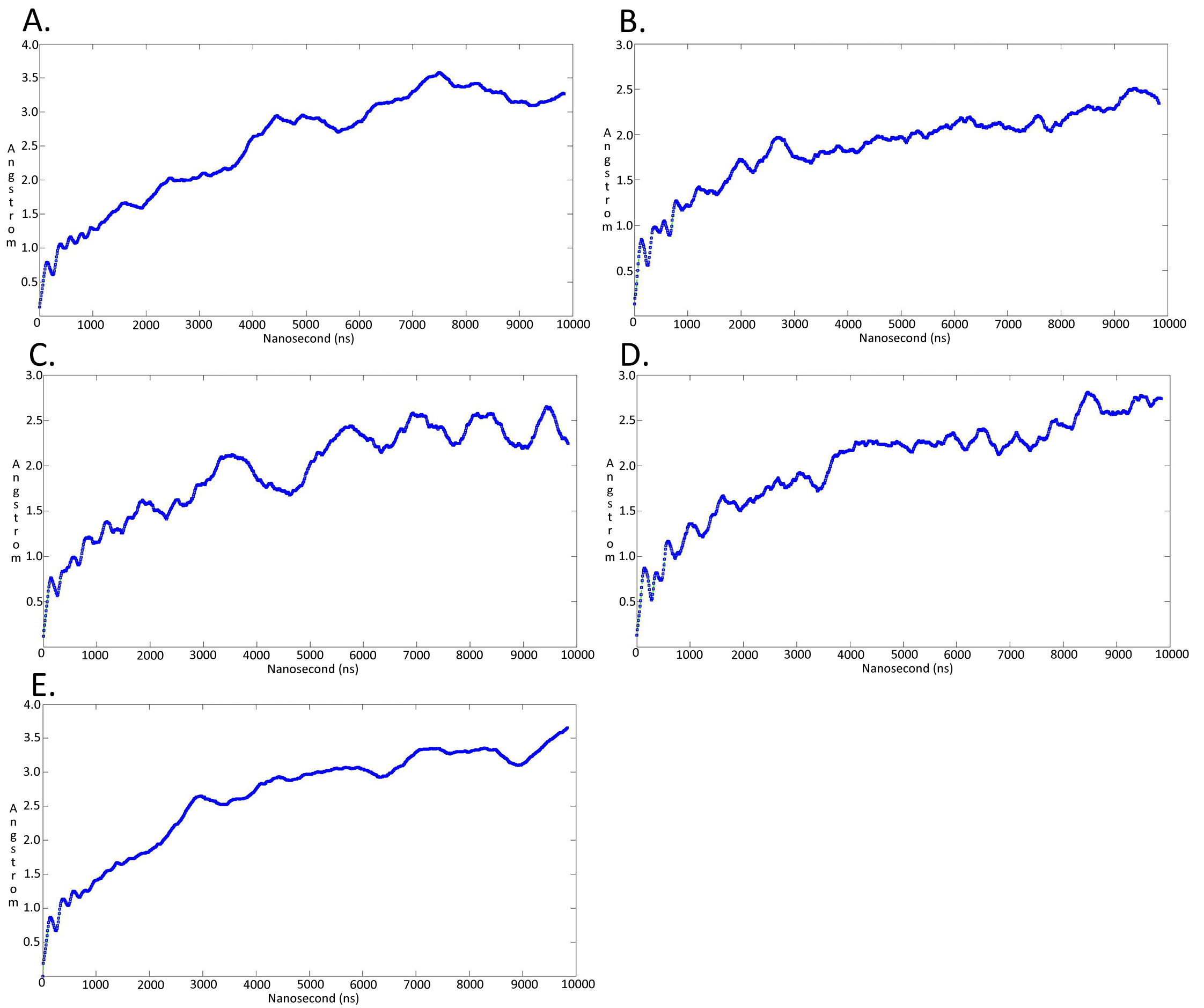

Supplement: Figure S6 — The energy (Kcal/mol) vs time (ns) plot of the 100ns simulation trajectory of the TBB DdRpII RPBI model B. Sub-domain regions of the Trypanosoma brucei brucei DdRPII RPB1 have been separated according to conventions of Fig. S3. (A) Domain A RMSD. (B) Domain B RMSD. (C) Domain C RMSD. (D) Domain D RMSD. (E) Domain E RMSD. [file peerj-05-3061-s006.png]

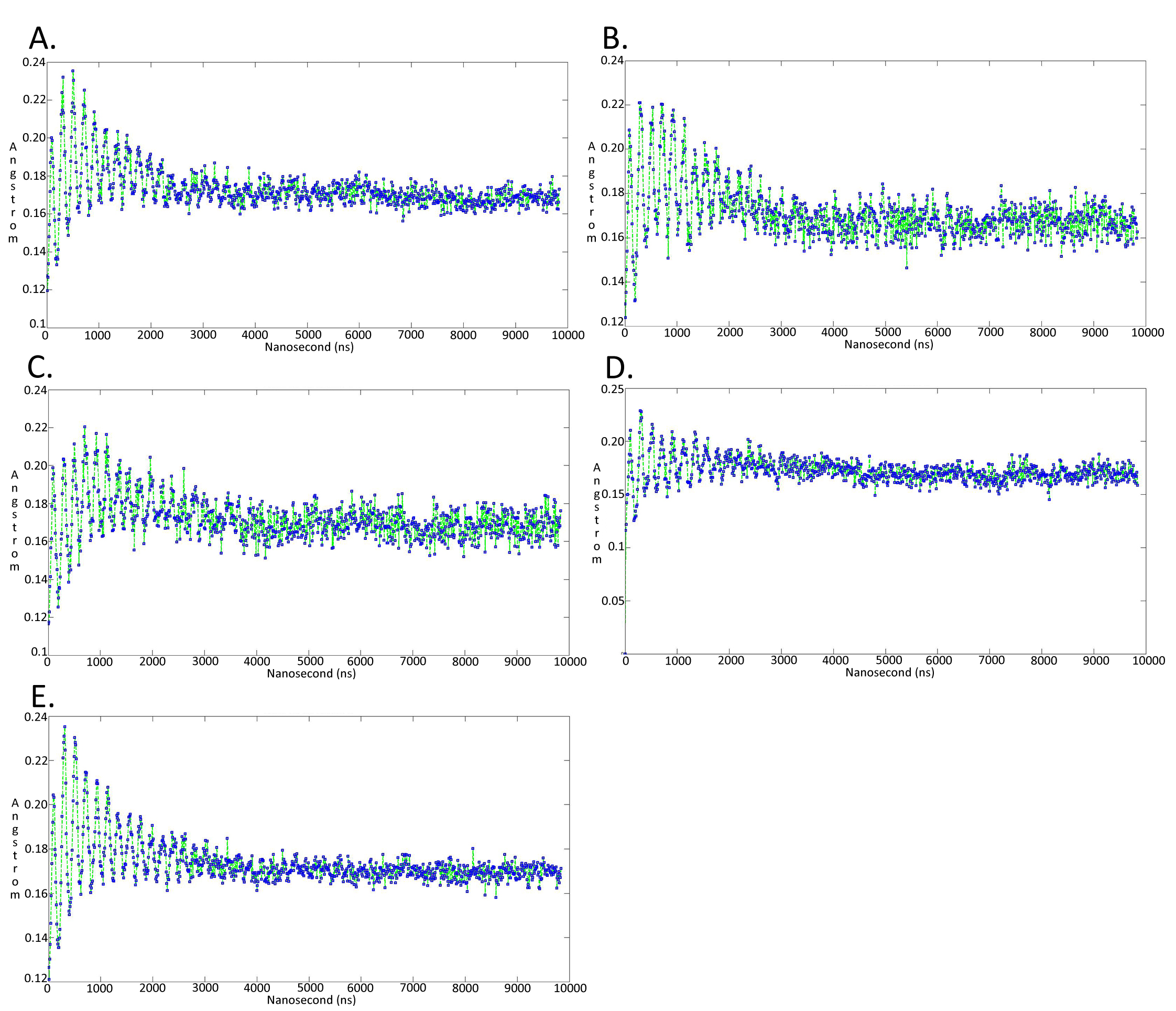

Supplement: Figure S7 — Sub-domain regions of the Trypanosoma brucei brucei DdRPII RPB1 have been separated according to conventions of Fig. S3. (A) Domain A RMSF. (B) Domain B RMSF. (C) Domain C RMSF. (D) Domain D RMSF. (E) Domain E RMSF. [file peerj-05-3061-s007.png]
