# Supplementary material for: Genetic and structural study of DNA-directed RNA polymerase II of Trypanosoma brucei, towards the designing of novel antiparasitic agents
Source: PeerJ. 2017 Mar 1;5:e3061. doi: 10.7717/peerj.3061 (PMC5335688; doi:10.7717/peerj.3061)

```
SC|1I3Q_A                 MVG-QQYS--SAPLRTVKEVQFGLFSPEEVRAISVAKIRFPETMDETQTR 47
HU|3J0K_A                 MVG-QQYS--SAPLRTVKEVQFGLFSPEEVRAISVAKIRFPETMDETQTR 47
SP|3H0G_A                 MSG-IQFSPSSVPLRRVEEVQFGILSPEEIRSMSVAKIEFPETMDESGQR 49
TBB|P17545                MSGGAALPVSQMELHKVNEVQFEIFKERQIKSYAVCLVEHAKSYANA--A 48
TBG|XP_011773113.1|DAL972 MSGGAALPVSQMELHKVNEVQFEIFKERQIKSYAVCLVEHAKSYERG--R 48
                          * *    .  .  *: *:**** ::. .:::: :*. :...::  .

                                              Motif 1A
SC|1I3Q_A                 AKIGGLNDPRLGS-IDRNLKCQTCQEGMNECPGHFGHIDLAKPVFHVGFI 96
HU|3J0K_A                 AKIGGLNDPRLGS-IDRNLKCQTCQEGMNECPGHFGHIDLAKPVFHVGFI 96
SP|3H0G_A                 PRVGGLLDPRLGT-IDRQFKCQTCGETMADCPGHFGHIELAKPVFHIGFL 98
TBB|P17545                DQSGEASMICVWVPLTSNSACETCHRKHPECPGHFGYIELAEPVFNIGVF 98
TBG|XP_011773113.1|DAL972 PVRGGINDLRMGT-TDFEFACETCHRKHPECPGHFGYIELAEPVFNIGVF 97
                             *      :      :  *:** .   :******:*:**:***::*.:
                                                   First Zinc Finger (Zn)

                                 Motif 2
SC|1I3Q_A                 AKIKKVCECVCMHCGKLLLDEHNELMRQALAIKDSKKRFAAIWTLCKTKM 146
HU|3J0K_A                 AKIKKVCECVCMHCGKLLLDEHNELMRQALAIKDSKKRFAAIWTLCKTKM 146
SP|3H0G_A                 SKIKKILECVCWNCGKLKIDSSNPKFNDTQRYRDPKNRLNAVWNVCKTKM 148
TBB|P17545                DLVLLVLKCVCKTCGALLLNTREQDVHKKLQHMTGLNRLRQVAKMAEAK- 147
TBG|XP_011773113.1|DAL972 DLVLLVLKCVCKTCGALLLNTREQDVHKKLQHMTGLNRLRQVAKMAEAK- 146
                            :  : :***  ** * ::  :  ...        :*:  : .:.::*

                                Second Zinc Finger (Zn)
SC|1I3Q_A                 VCETDVPSEDD------PTQLVSRG---------GCGNTQPTIRKDGLKL 181
HU|3J0K_A                 VCETDVPSEDD------PTQLVSRG---------GCGNTQPTIRKDGLKL 181
SP|3H0G_A                 VCDTGLSAGSDNFDLSNPSANMGHG---------GCGAAQPTIRKDGLRL 189
TBB|P17545                -CRVSTSTEDDMGIDGFDSAPFNGGSGMGPGATRGCGASQPRVS----RF 192
TBG|XP_011773113.1|DAL972 -CRVSTSTEDDMGIDGFDSAPFNGGSGMGPGATRGCGASQPRVS----RF 191
                           * .. .: .*       :  .. *         *** :** :     ::
                                                          Second Zinc Finger (Zn)

SC|1I3Q_A                 VGSWKKDRATGDADEPELRVLSTEEILNIFKHISVKDFTSLGFNEVFSRP 231
HU|3J0K_A                 VGSWKKDRATGDADEPELRVLSTEEILNIFKHISVKDFTSLGFNEVFSRP 231
SP|3H0G_A                 WGSWK--RGKDESDLPEKRLLSPLEVHTIFTHISSEDLAHLGLNEQYARP 237
TBB|P17545                YGIYPTLVIKAVHEEQD-AEWHADKVRQVLDRVSDDDARLMGFDPQRCHP 241
TBG|XP_011773113.1|DAL972 YGIYPTLVIKAVHEEQD-AEWHADKVRQVLDRVSDDDARLMGFDPQRCHP 240
                           * :     .   :  :     . ::  :: ::* .*   :*::   .:*

                                  Motif 1B
SC|1I3Q_A                 EWMILTCLPVPPPPVRPSISFNESQRGEDDLTFKLADILKANISLETLEH 281
HU|3J0K_A                 EWMILTCLPVPPPPVRPSISFNESQRGEDDLTFKLADILKANISLETLEH 281
SP|3H0G_A                 DWMIITVLPVPPPSVRPSISVDGTSRGEDDLTHKLSDIIKANANVRRCEQ 287
TBB|P17545                RDLVLTVLPVPPPQVRPAISFGG-LRSDDELTHQIMSIVKRNNQLRR-DK 289
TBG|XP_011773113.1|DAL972 RDLVLTVLPVPPPQVRPAISFGG-LRSDDELTHQIMSIVKRNNQLRR-DK 288
                            :::* ****** ***:**..   *.:*:**.:: .*:* * .:.  ::
                                                   Dock

SC|1I3Q_A                 NGAPHHAIEEAESLLQFHVATYMDNDIAGQPQALQKSGRPVKSIRARLKG 331
HU|3J0K_A                 NGAPHHAIEEAESLLQFHVATYMDNDIAGQPQALQKSGRPVKSIRARLKG 331
SP|3H0G_A                 EGAPAHIVSEYEQLLQFHVATYMDNEIAGQPQALQKSGRPLKSIRARLKG 337
TBB|P17545                ESDVQAAIDRSRALLQEHVATYFNNASTYYKPTKVNDTKKLKSLTERLKG 339
TBG|XP_011773113.1|DAL972 ESDVQAAIDRSRALLQEHVATYFNNASTYYKPTKVNDTKKLKSLTERLKG 338
                          :.     :.. . *** *****::*  :    :  :. : :**:  ****

                                       Motif 3A
SC|1I3Q_A                 KEGRIRGNLMGKRVDFSARTVISGDPNLELDQVGVPKSIAKTLTYPEVVT 381
HU|3J0K_A                 KEGRIRGNLMGKRVDFSARTVISGDPNLELDQVGVPKSIAKTLTYPEVVT 381
SP|3H0G_A                 KEGRLRGNLMGKRVDFSARTVITGDPNLSLDELGVPRSIAKTLTYPETVT 387
TBB|P17545                KYGRLRGNLMGKRVDFSARTVITGDPNIDVDEVGVPFSVAMTLTFPERVN 389
TBG|XP_011773113.1|DAL972 KYGRLRGNLMGKRVDFSARTVITGDPNIDVDEVGVPFSVAMTLTFPERVN 388
                          * **:*****************:****:.:*::*** *:* ***:** *.

SC|1I3Q_A                 PYNIDRLTQLVRNGPNEHPGAKYVIRDSGDRIDLRYSKRAGDIQLQYGWK 431
HU|3J0K_A                 PYNIDRLTQLVRNGPNEHPGAKYVIRDSGDRIDLRYSKRAGDIQLQYGWK 431
SP|3H0G_A                 PYNIYQLQELVRNGPDEHPGAKYIIRDTGERIDLRYHKRAGDIPLRYGWR 437
TBB|P17545                TINKKRLTEFARR--TVYPSANYIHHPNGTITKLALLRDRSKVTLNIGDV 437
TBG|XP_011773113.1|DAL972 TVNKKRLTEFARR--TVYPSANYIHHPNGTITKLALLRDRSKVTLNIGDV 436
                          . *  :* ::.*.    :*.*:*: : .*   .*   :  ..: *. *
```

CLAMP CORE

CLAMP HEAD

CLAMP CORE

ACTIVE SITE

Dock

```
                    Motif 3B
SC|1I3Q_A                    VERHIMDNDPVLFNRQPSLHKMSMMAHRVKVIPYSTFRLNLSVTSPYNAD 481
HU|3J0K_A                    VERHIMDNDPVLFNRQPSLHKMSMMAHRVKVIPYSTFRLNLSVTSPYNAD 481
SP|3H0G_A                    VERHIRDGDVVIFNRQPSLHKMSMMGHRIRVMPYSTFRLNLSVTSPYNAD 487
TBB|P17545                   VERHVINGDVVLFNRQPTLHRMSMMGHRVRVLNYNTFRLNLSCTTPYNAD 487
TBG|XP_011773113.1|DAL972    VERHVINGDVVLFNRQPTLHRMSMMGHRVRVLNYNTFRLNLSCTTPYNAD 486
                             ****: :.* *:*****:**:****.**::*: *.******* *:*****

                    Motif 3C
SC|1I3Q_A                    FDGDEMNLHVPQSEETRAELSQLCAVPLQIVSPQSNKPCMGIVQDTLCGI 531
HU|3J0K_A                    FDGDEMNLHVPQSEETRAELSQLCAVPLQIVSPQSNKPCMGIVQDTLCGI 531
SP|3H0G_A                    FDGDEMNMHVPQSEETRAEIQEITMVPKQIVSPQSNKPVMGIVQDTLAGV 537
TBB|P17545                   FDGDEMNLHVPQSLLTKAELIEMMMVPKNFVSPNKSAPCMGIVQDSLLGS 537
TBG|XP_011773113.1|DAL972    FDGDEMNLHVPQSLLTKAELIEMMMVPKNFVSPNKSAPCMGIVQDSLLGS 536
                             *******:*****  *:**: ::  ** ::***:.. * ******:* *
                    METAL A

SC|1I3Q_A                    RKLTLRDTFIELDQVLNMLYWVPDWDGVIPTPAIIKPKPLWSGKQILSVA 581
HU|3J0K_A                    RKLTLRDTFIELDQVLNMLYWVPDWDGVIPTPAIIKPKPLWSGKQILSVA 581
SP|3H0G_A                    RKFSLRDNFLTRNAVMNIMLWVPDWDGILPPPVILKPKVLWTGKQILSLI 587
TBB|P17545                   YRLTDKDTFLDKYFVQSVALWLDLWQ--LPIPAILKPRPLWTGKQVFSLI 585
TBG|XP_011773113.1|DAL972    YRLTDKDTFLDKYFVQSVALWLDLWQ--LPIPAILKPRPLWTGKQVFSLI 584
                              ::: :*.*:    * .:  *:  *:  :* *.*:**: **:***::*:

SC|1I3Q_A                    IPNGIHLQRFDEGTTLLSPKDNGMLIIDGQIIFGVVEKKTVGSSNGGLIH 631
HU|3J0K_A                    IPNGIHLQRFDEGTTLLSPKDNGMLIIDGQIIFGVVEKKTVGSSNGGLIH 631
SP|3H0G_A                    IPKGINLIRDDDKQSLSNPTDSGMLIENGEIIYGVVDKKTVGASQGGLVH 637
TBB|P17545                   LPEVNHPATPQDRPPFPHN-DSVVMIRRGQLLCGPITKSIVGAAPGSLIH 634
TBG|XP_011773113.1|DAL972    LPEVNHPATPQDRPPFPHN-DSVVMIRRGQLLCGPITKSIVGAAPGSLIH 633
                             :*:  :    ::  .:    *. ::*  *::: * : *. **:: *.*:*

SC|1I3Q_A                    VVTREKGPQVCAKLFGNIQKVVNFWLLHNGFSTGIGDTIADGPTMREITE 681
HU|3J0K_A                    VVTREKGPQVCAKLFGNIQKVVNFWLLHNGFSTGIGDTIADGPTMREITE 681
SP|3H0G_A                    TIWKEKGPEICKGFFNGIQRVVNYWLLHNGFSIGIGDTIADADTMKEVTR 687
TBB|P17545                   VIFNEHGSDEVARFINGVQRVTTFFLLNFGFSVGVQDTVADSDTLRQMND 684
TBG|XP_011773113.1|DAL972    VIFNEHGSDEVARFINGVQRVTTFFLLNFGFSVGVQDTVADSDTLRQMND 683
                             .: .*:*.:    ::..:*:*..::**: *** *: **:**. *::::.

SC|1I3Q_A                    TIAEAKKKVLDVTKEAQANLLTAKHGMTLRESFEDNVVRFLNEARDKAGR 731
HU|3J0K_A                    TIAEAKKKVLDVTKEAQANLLTAKHGMTLRESFEDNVVRFLNEARDKAGR 731
SP|3H0G_A                    TVKEARRQVAECIQDAQHNRLKPEPGMTLRESFEAKVSRILNQARDNAGR 737
TBB|P17545                   VLVKTRRNVEKIGAAANNRTLNRKAGMTLLQSFEADVNSALNKCREEAAK 734
TBG|XP_011773113.1|DAL972    VLVKTRRNVEKIGAAANNRTLNRKAGMTLLQSFEADVNSALNKCREEAAK 733
                             .: :::::* .    *: . *. : **** :*** .*   **:.*::*.:

SC|1I3Q_A                    LAEVNLKDLNNVKQMVMAGSKGSFINIAQMSACVGQQSVEGKRIAFGFVD 781
HU|3J0K_A                    LAEVNLKDLNNVKQMVMAGSKGSFINIAQMSACVGQQSVEGKRIAFGFVD 781
SP|3H0G_A                    SAEHSLKDSNNVKQMVAAGSKGSFINISQMSACVGQQIVEGKRIPFGFKY 787
TBB|P17545                   KALSNVRRTNSFKVMIEAGSKGTDLNICQIAVFVGQQNVAGSRIPFGFRR 784
TBG|XP_011773113.1|DAL972    KALSNVRRTNSFKVMIEAGSKGTDLNICQIAVFVGQQNVAGSRIPFGFRR 783
                              *  .::  *..* *: *****: :**.*::. **** * *.**.***

                                                              Motif 4A   Motif 4B
SC|1I3Q_A                    RTLPHFSKDDYSPESKGFVENSYLRGLTPQEFFFHAMGGREGLIDTAVKT 831
HU|3J0K_A                    RTLPHFSKDDYSPESKGFVENSYLRGLTPQEFFFHAMGGREGLIDTAVKT 831
SP|3H0G_A                    RTLPHFPKDDDSPESRGFIENSYLRGLTPQEFFFHAMAGREGLIDTAVKT 837
TBB|P17545                   RTLPHFMLDDYGETSRGMANRGYVEGLKPHEFFFHTMAGREGLIDTAVKT 834
TBG|XP_011773113.1|DAL972    RTLPHFMLDDYGETSRGMANRGYVEGLKPHEFFFHTMAGREGLIDTAVKT 833
                             ******  ** .  *:*: :..*:.**.*:*****:*.************

SC|1I3Q_A                    AETGYIQRRLVKALEDIMVHYDNTTRNSLGNVIQFIYGEDGMDAAHIEKQ 881
HU|3J0K_A                    AETGYIQRRLVKALEDIMVHYDNTTRNSLGNVIQFIYGEDGMDAAHIEKQ 881
SP|3H0G_A                    AETGYIQRRLVKAMEDVMVRYDGTVRNAMGDIIQFAYGEDGLDATLVEYQ 887
TBB|P17545                   SDTGYLQRKLIKALEDVHAAYDGTVRNANDELIQFMYGEDGLDGARIEGG 884
TBG|XP_011773113.1|DAL972    SDTGYLQRKLIKALEDVHAAYDGTVRNANDELIQFMYGEDGLDGARIEGG 883
                             ::***:**:*:**:**: . **.*.**: .::*** *****:*.: :*
```

ACTIVE SITE

PORE 1

FUUNEL

CLEFT

```
SC|1I3Q_A                 SLDTIGGSD-----AAFEKRYRVDLLNTDHTLDPSLLESG-SEILGDLKL 925
HU|3J0K_A                 SLDTIGGSD-----AAFEKRYRVDLLNTDHTLDPSLLESG-SEILGDLKL 925
SP|3H0G_A                 VFDSLRLST-----KQFEKKYRIDLME-DRSLSLYMENS----IENDSSV 927
TBB|P17545                QLFPLPFRDDKEMEDTYKYEYDVDGTFSGKVGGNYMDPHVRKMLRADPQN 934
TBG|XP_011773113.1|DAL972 QLFPLPFRDDKEMEDTYKYEYDVDGTFSGKVGGNYMDPHVRKMLRADPQN 933
                           : .:           :: .* :*    .:  .  :       :  * .

SC|1I3Q_A                 QVLLDEEYKQLVKDRKFLR-EVFVDG--EANWPLPVNIRRIIQNAQQTFH 972
HU|3J0K_A                 QVLLDEEYKQLVKDRKFLR-EVFVDG--EANWPLPVNIRRIIQNAQQTFH 972
SP|3H0G_A                 QDLLDEEYTQLVADRELLCKFIFPKG--DARWPLPVNVQRIIQNALQIFH 975
TBB|P17545                VRKLQEEYEQLTADREWSRKMLDLEDRDKLKLNLPVNPGRLIQNARSTMG 984
TBG|XP_011773113.1|DAL972 VRKLQEEYEQLTADREWSRKMLDLEDRDKLKLNLPVNPGRLIQNARSTMG 983
                             *:*** **. **:     :  ..  . .  ****  *:**** . :

SC|1I3Q_A                 IDHTKPSDLTIKDIVLGVKDLQENLLVLRG----------KNEIIQNAQR 1012
HU|3J0K_A                 IDHTKPSDLTIKDIVLGVKDLQENLLVLRG----------KNEIIQNAQR 1012
SP|3H0G_A                 LEAKKPTDLLPSDIINGLNELIAKLTIFRG----------SDRITRDVQN 1015
TBB|P17545                KRSQ-VSNLSPITIIDHVRKLQEDLMKLFPSYHRGGDGYIRNTLSRERIE 1033
TBG|XP_011773113.1|DAL972 KRSQ-VSNLSPITIIDHVRKLQEDLMKLFPSYHRGGDGYIRNTLSRERIE 1032
                                ::*    *:  :..*  .*  :             : : ::  .

SC|1I3Q_A                 DAVTLFCCLLRSRLATRRVLQEYRLTKQAFDWVLSNIEAQFLRSVVHPGE 1062
HU|3J0K_A                 DAVTLFCCLLRSRLATRRVLQEYRLTKQAFDWVLSNIEAQFLRSVVHPGE 1062
SP|3H0G_A                 NATLLFQILLRSKFAVKRVIMEYRLNKVAFEWIMGEVEARFQQAVVSPGE 1065
TBB|P17545                SALTLFNVHLRQLLASKRVLKEYKLNDRAFEYLLKEIRTKYHQSLTTPGE 1083
TBG|XP_011773113.1|DAL972 SALTLFNVHLRQLLASKRVLKEYKLNDRAFEYLLKEIRTKYHQSLTTPGE 1082
                          .*  **   **. :* :**: **:*.. **:::: ::.::: :::. ***

                                    Motif 4C
SC|1I3Q_A                 MVGVLAAQSIGEPATQMTLNTFHFAGVASKKVTSGVPRLKEILNVAKNMK 1112
HU|3J0K_A                 MVGVLAAQSIGEPATQMTLNTFHFAGVASKKVTSGVPRLKEILNVAKNMK 1112
SP|3H0G_A                 MVGTLAAQSIGEPATQMTLNTFHYAGVSSKNVTLGVPRLKEILNVAKNIK 1115
TBB|P17545                NIGAIAAQSCGEPATQMTLNTFHNAGISSKNVTLGVPRLLELLNVSRNQK 1133
TBG|XP_011773113.1|DAL972 NIGAIAAQSCGEPATQMTLNTFHNAGISSKNVTLGVPRLLELLNVSRNQK 1132
                           :*.:**** ************* **::**:** ***** *:***::* *

SC|1I3Q_A                 TPSLTVYLEPGHAADQEQAKLIRSAIEHTTLKSVTIASEIYYDPDPRSTV 1162
HU|3J0K_A                 TPSLTVYLEPGHAADQEQAKLIRSAIEHTTLKSVTIASEIYYDPDPRSTV 1162
SP|3H0G_A                 TPSLTIYLMPWIAANMDLAKNVQTQIEHTTLSTVTSATEIHYDPDPQDTV 1165
TBB|P17545                HASMTVSLFPPYDEKRN-AQKAQHLIEYCTLESITRRIQFIYDPDPRHTV 1182
TBG|XP_011773113.1|DAL972 HASMTVSLFPPYDEKRN-AQKAQHLIEYCTLESITRRIQFIYDPDPRHTV 1181
                           .*:*: * *    . : *:  :  **: **.::*   :: *****: **

SC|1I3Q_A                 IPEDEEIIQLHFSLLDEE----AEQSFDQQSPWLLRLELDRAAMNDKDLT 1208
HU|3J0K_A                 IPEDEEIIQLHFSLLDEE----AEQSFDQQSPWLLRLELDRAAMNDKDLT 1208
SP|3H0G_A                 IEEDKDFVEAFFAIPDEE----VEENLYKQSPWLLRLELDRAKMLDKKLS 1211
TBB|P17545                VEADRDILELEWNVMDESDAELRIQEVVAGSPWVVRLELDVDMVTDKALD 1232
TBG|XP_011773113.1|DAL972 VEADRDILELEWNVMDESDAELRIQEVVAGSPWVVRLELDVDMVTDKALD 1231
                          :  *.::::  : : **.      :..   ***::*****   : ** *

SC|1I3Q_A                 MGQVGERIKQTFKNDLFVIWSEDNDEKLIIRCRVVRP---KSLDAETEAE 1255
HU|3J0K_A                 MGQVGERIKQTFKNDLFVIWSEDNDEKLIIRCRVVRP---KSLDAETEAE 1255
SP|3H0G_A                 MSDVAGKIAESFERDLFTIWSEDNADKLIIRCRIIRDDDRKAEDDDNMIE 1261
TBB|P17545                MKDVKQAILRVDESYIIETGMANNVRQRTIRMRSRYN---------EGAD 1273
TBG|XP_011773113.1|DAL972 MKDVKQAILRVDESYIIETGMANNVRQRTIRMRSRYN---------EGAD 1272
                          * :*   * .  :  ::     :*  :  ** *                :

SC|1I3Q_A                 EDHMLKKIENTMLENITLRGVENIERVVMMKY-DRKVPSPTGEYVKEPEW 1304
HU|3J0K_A                 EDHMLKKIENTMLENITLRGVENIERVVMMKY-DRKVPSPTGEYVKEPEW 1304
SP|3H0G_A                 EDVFLKTIEGHMLESISLRGVPNITRVYMMEH-KIVRQIEDGTFERADEW 1310
TBB|P17545                SIPKLKREIPALLARVHLRGIPGVRRALLKDTTEFTVDQATGKMSGNKIW 1323
TBG|XP_011773113.1|DAL972 SIPKLKREIPALLARVHLRGIPGVRRALLKDTTEFTVDQATGKMSGNKIW 1322
                          .   **     :*  : ***: .: *. : .  .       *       *
```

FOOT

CLEFT

JAW

```
SC|1I3Q_A                       VLETDGVNLSEVMT-VPG------IDPTRIYTNSFIDIMEVLGIEAGRAA 1347
HU|3J0K_A                       VLETDGVNLSEVMT-VPG------IDPTRIYTNSFIDIMEVLGIEAGRAA 1347
SP|3H0G_A                       VLETDGINLTEAMT-VEG------VDATRTYSNSFVEILQILGIEATRSA 1353
TBB|P17545                      AIDTDGTALRRAFIGVVGEDGKNIINAVKTSSNKVPEVCSLLGIEAARSK 1373
TBG|XP_011773113.1|DAL972       AIDTDGTALRRAFIGVVGEDGKNIINAVKTSSNKVPEVCSLLGIEAARSK 1372
                                .::***  *  ..:  * *      ::..:  :*.. :: .:***** *:

SC|1I3Q_A                       LYKEVYNVIASDGSYVNYRHMALLVDVMTTQGGLTSVTRHGFNRSNT-GA 1396
HU|3J0K_A                       LYKEVYNVIASDGSYVNYRHMALLVDVMTTQGGLTSVTRHGFNRSNT-GA 1396
SP|3H0G_A                       LLKELRNVIEFDGSYVNYRHLALLCDVMTSRGHLMAITRHGINRAET-GA 1402
TBB|P17545                      MLTELREAYLAYGLNINYRHYTILVDTICQHGYLMAVSRSGINRSDTSGP 1423
TBG|XP_011773113.1|DAL972       MLTELREAYLAYGLNINYRHYTILVDTICQHGYLMAVSRSGINRSDTSGP 1422
                                : .*: :.    *  :**** ::* *.:  :* * :::* *:**::* *.

SC|1I3Q_A                       LMRCSFEETVEILFEAGASAELDDCRGVSENVILGQMAPIGTGAFDVMID 1446
HU|3J0K_A                       LMRCSFEETVEILFEAGASAELDDCRGVSENVILGQMAPIGTGAFDVMID 1446
SP|3H0G_A                       LMRCSFEETVEILMDAAASGEKDDCKGISENIMLGQLAPMGTGAFDIYLD 1452
TBB|P17545                      LMRCSFEETVKVLMAAAASFGECDPVRGVSANLVLGNQARVGTGLFDLVLN 1473
TBG|XP_011773113.1|DAL972       LMRCSFEETVKVLMAAAASFGECDPVRGVSANLVLGNQARVGTGLFDLVLN 1472
                                **********::*: *.: .* *  :*:* *::**: * :*** **: ::

SC|1I3Q_A                       EESLVKYMPEQKITEIEDG-------QDGGVTPYSNESGLVNADLDVKDE 1489
HU|3J0K_A                       EESLVKYMP----------------------------------------- 1455
SP|3H0G_A                       QDMLMNYSLGTAVPTLAGSGMGTSQLPEGAGTPYERSPMVDSGFVGSPDA 1502
TBB|P17545                      MAALQQAVPQAEAVAPGKDVNVYHSLGSTLQQNIQSSIAYRPRDHDATPF 1523
TBG|XP_011773113.1|DAL972       MAALQQAVPQAEAVAPGKDVNVYHSLGSTLQQNIQSSIAYRPRDHDATPF 1522
```

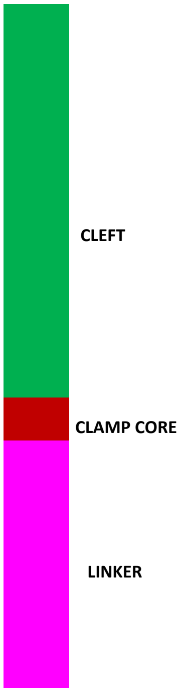

Supplement: Figure S2 — The alignment was performed using the Trypanosoma brucei brucei DdRPII RPB1, the Trypanosoma brucei gambiense DdRpII RPB1, the crystal structure of Schizosaccharomyces pombe DdRpII RPB, the crystal structure of Saccharomyces cerevisiae DdRpII RPB1 and the electron microscopy structure of Human DdRpII DdRpII RPB1. (A) All nine suggested conserved motifs and major domains of DdRpII RPB1 have been marked (Motifs 1a, 1b, 2, 3a, 3b, 3c, 4a, 4b, 4c). Additionally, in the multiple sequence alignment were presented the major differences. (B) Domains and domainlike regions of the DdRpII subunit Rpb1. The amino acid residue numbers at the domain boundaries are indicated. [file peerj-05-3061-s002.pdf]
