# Supplementary material for: Genetic and structural study of DNA-directed RNA polymerase II of Trypanosoma brucei, towards the designing of novel antiparasitic agents
Source: PeerJ. 2017 Mar 1;5:e3061. doi: 10.7717/peerj.3061 (PMC5335688; doi:10.7717/peerj.3061)
Supplement: Figure S3 — The alignment was performed using the Trypanosoma brucei brucei DdRPII RPB1, the crystal structure of Schizosaccharomyces pombe DdRpII RPB and the electron microscopy structure of Bos taurus DdRpII RPB1. All five sub-domains (A-E) as referred in Pfam database have been marked with different colours. [file peerj-05-3061-s003.pdf]

```
gi|133332|RPB1A_TRYBB       -MSGGAALPVSQMELHKVNEVQFEIFKERQIKSYAVCLVEHAKSYANAADQSGEASMICV
3H0G:A|PDBID|S. Pombe       --MSGIQFSPSSVPLRRVEEVQFGILSPEEIRSMSVA----KIEFPETMDESGQRPRVGG
5FLM:A|PDBID|Bos Taurus     MHGGGPPSGDSACPLRTIKRVQFGVLSPDELKRMSVT--EGGIKYPETTE--GGRPKLGG
                               .*     *   *. :: *** ::.  ::.  :*       .:.:: :  *  . :

gi|133332|RPB1A_TRYBB       WVP-----LTSNSACETCHRKHPECPGHFGYIELAEPVFNIGVFDLVLLVLKCVCKTCGA
3H0G:A|PDBID|S. Pombe       LLDPRLGTIDRQFKCQTCGETMADCPGHFGHIELAKPVFHIGFLSKIKKILECVCWNCGK
5FLM:A|PDBID|Bos Taurus     LMDPRQGVIERTGRCQTCAGNMTECPGHFGHIELAKPVFHVGFLVKTMKVLRCVCFFCSK
                             :      :     *:**  . .:******:****:***::*.:     :* ***  *.

gi|133332|RPB1A_TRYBB       LLLNTREQDVHKKLQHVTGLNRLRQVAKMAEAKCRVSTSTEDDMGIDGFDSAPFNGGSGM
3H0G:A|PDBID|S. Pombe       LKIDSSNPKFND--TQRYRDPKNRLNAVWNVCKTKMVCDTGLSAGSDNFDLSNPSAN--M
5FLM:A|PDBID|Bos Taurus     LLVDSNNPKIKDILAKSKGQPKKRLTHVYDLCKGKNICEGGEEMD-NKFGVEQPEGDEDL
                            * ::: : ..:.   :     . *       .* .   .   . . : *.    ...  :

gi|133332|RPB1A_TRYBB       ----GPGATRGCGASQPRVSR----FYGIYPTLVIKAVHEEQDAE----WHADKVRQVLD
3H0G:A|PDBID|S. Pombe       ----GHG---GCGAAQPTIRKDGLRLWGSW-----KRGKDESDLPEKRLLSPLEVHTIFT
5FLM:A|PDBID|Bos Taurus     TKEKGHG---GCGRYQPRIRRSGLELYAEW-----KHVNEDSQ-EKKILLSPERVHEIFK
                                * *   ***  ** : .    ::. :     *  :::.:        .  *. ::

gi|133332|RPB1A_TRYBB       RVSDDDARLMGFDPQRCHPRDLVLTVLPVPPPQVRPAISF-GGLRSDDELTHQIMSIVKR
3H0G:A|PDBID|S. Pombe       HISSEDLAHLGLNEQYARPDWMIITVLPVPPPSVRPSISVDGTSRGEDDLTHKLSDIIKA
5FLM:A|PDBID|Bos Taurus     RISDEECFVLGMEPRYARPEWMIVTVLPVPPLSVRPAVVMQGSARNQDDLTHKLADIVKI
                            .:*.::   :*:: . ..*  :::******* .***:: . *  *.:*:***:: .*:*

gi|133332|RPB1A_TRYBB       NNQLRRDKESDVQA-AIDRSRALLQEHVATYFNNASTYYKPTKVNDTKKLKSLTERLKGK
3H0G:A|PDBID|S. Pombe       NANVRRCEQEGAPAHIVSEYEQLLQFHVATYMDNEIAGQPQALQKSGRPLKSIRARLKGK
5FLM:A|PDBID|Bos Taurus     NNQLRRNEQNGAAAHVIAEDVKLLQFHVATMVDNELPGLPRAMQKSGRPLKSLKQRLKGK
                            * ::** ::... *  :     *** **** .:*  .    :  :. . ***:  *****

gi|133332|RPB1A_TRYBB       YGRLRGNLMGKRVDFSARTVITGDPNIDVDEVGVPFSVAMTLTFPERVNTVNKKRLTEFA
3H0G:A|PDBID|S. Pombe       EGRLRGNLMGKRVDFSARTVITGDPNLSLDELGVPRSIAKTLTYPETVTPYNIYQLQELV
5FLM:A|PDBID|Bos Taurus     EGRVRGNLMGKRVDFSARTVITPDPNLSIDQVGVPRSIAANMTFAEIVTPFNIDRLQELV
                             **:****************** ***:.:*::*** *:* .:*:.* *.. *  .* *:.

gi|133332|RPB1A_TRYBB       RR--TVYPSANYIHHPNGTITKLALLRDRSKVTLNIGDVVERHVINGDVVLFNRQPTLHR
3H0G:A|PDBID|S. Pombe       RNGPDEHPGAKYIIRDTGERIDLRYHKRAGDIPLRYGWRVERHIRDGDVVIFNRQPSLHK
5FLM:A|PDBID|Bos Taurus     RRGNSQYPGAKYIIRDNGDRIDLRFHPKPSDLHLQTGYKVERHMCDGDIVIFNRQPTLHK
                            *.    :*.*:** . .*   .*      ..: *. *  ****: :**:*:*****:**.

gi|133332|RPB1A_TRYBB       MSMMGHRVRVLNYSTFRLNLSCTTPYNADFDGDEMNLHVPQSLLTKAELIEMMMVPKNFV
3H0G:A|PDBID|S. Pombe       MSMMGHRIRVMPYSTFRLNLSVTSPYNADFDGDEMNMHVPQSEETRAEIQEITMVPKQIV
5FLM:A|PDBID|Bos Taurus     MSMMGHRVRILPWSTFRLNLSVTTPYNADFDGDEMNLHLPQSLETRAEIQELAMVPRMIV
                            *******:*:: :******** *:************:*:***  *.**: *: ***. :*

gi|133332|RPB1A_TRYBB       SPNKSAPCMGIVQDSLLGSYRLTDKDTFLDKYFVQSVALWLDLWQ--LPIPAILKPRPLW
3H0G:A|PDBID|S. Pombe       SPQSNKPVMGIVQDTLAGVRKFSLRDNFLTRNAVMNIMLWVPDWDGILPPPVILKPKVLW
5FLM:A|PDBID|Bos Taurus     TPQSNRPVMGIVQDTLTAVRKFTKRDVFLERGEVMNLLMFLSTWDGKVPQPAILKPRPLW
                            :*:.. * ******:* .  .:: .* ** .  * .: :::  *:  :* *.****. **

gi|133332|RPB1A_TRYBB       TGKQVFSLILPEVNHPAT-------PQDRPPFPH---NDSVVMIRRGQLLCGPITKSIVG
3H0G:A|PDBID|S. Pombe       TGKQILSLIIPKGINLIR-------DDDKQSLSN--PTDSGMLIENGEIIYGVVDKKTVG
5FLM:A|PDBID|Bos Taurus     TGKQIFSLIIPGHINCIRTHSTHPDDEDSGPYKHISPGDTKVVVENGELIMGILCKKSLG
                            ****::***:*   :           :*  .  :    *: ::: .*::: * : *. :*

gi|133332|RPB1A_TRYBB       AAPGSLIHVIFNEHGSDEVARFINGVQRVTTFFLLNFGFSVGVQDTVADSDTLRQMNDVL
3H0G:A|PDBID|S. Pombe       ASQGGLVHTIWKEKGPEICKGFFNGIQRVVNYWLLHNGFSIGIGDTIADADTMKEVTRTV
5FLM:A|PDBID|Bos Taurus     TSAGSLVHISYLEMGHDITRLFYSNIQTVINNWLLIEGHTIGIGDSIADSKTYQDIQNTI
                            :: *.*:*  : * * :    * ..:* * . :**  *.::*: *::**:.* .::  .:

gi|133332|RPB1A_TRYBB       VKTRRNVEKIGAAANNRTLNRKAGMTLLQSFEADVNSALNKCREEAAKKALSNVRRTNSF
3H0G:A|PDBID|S. Pombe       KEARRQVAECIQDAQHNRLKPEPGMTLRESFEAKVSRILNQARDNAGRSAEHSLKDSNNV
5FLM:A|PDBID|Bos Taurus     KKAKQDVIEVIEKAHNNELEPTPGNTLRQTFENQVNRILNDARDKTGSSAQKSLSEYNNF
                             ::..:* :    *::. *:  .* ** ::** .*.  **..*:::. .*  .:   *..

gi|133332|RPB1A_TRYBB       KVMIEAGSKGTDLNICQIAVFVGQQNVAGSRIPFGFRRRTLPHFMLDDYGETSRGMANRG
3H0G:A|PDBID|S. Pombe       KQMVAAGSKGSFINISQMSACVGQQIVEGKRIPFGFKYRTLPHFPKDDDSPESRGFIENS
5FLM:A|PDBID|Bos Taurus     KSMVVSGAKGSKINISQVIAVVGQQNVEGKRIPFGFKHRTLPHFIKDDYGPESRGFVENS
                            * *: :*:**: :**.*: . **** * *.******. ******  **  .  ***: :..

gi|133332|RPB1A_TRYBB       YVEGLKPHEFFFHTMAGREGLIDTAVKTSDTGYLQRKLIKALEDVHAAYDGTVRNANDEL
3H0G:A|PDBID|S. Pombe       YLRGLTPQEFFFHAMAGREGLIDTAVKTAETGYIQRRLVKAMEDVMVRYDGTVRNAMGDI
5FLM:A|PDBID|Bos Taurus     YLAGLTPTEFFFHAMGGREGLIDTAVKTAETGYIQRRLIKSMESVMVKYDATVRNSINQV
                            *: **.* *****:*.************::***:**.*:*::*.* . **.****: .::

gi|133332|RPB1A_TRYBB       IQFMYGEDGLDGARIEGGQLFPLPFRDDKEMEDTYKYEYDVDGTFSGKVGGNYMDPHVRK
3H0G:A|PDBID|S. Pombe       IQFAYGEDGLDATLVEYQVFDSLRL-STKQFEKKYRIDLMEDRSLS-----LYMENSIEN
5FLM:A|PDBID|Bos Taurus     VQLRYGEDGLAGESVEFQNLATLKP-SNKAFEKKFRFDYTNERALR-----RTLQEDLVK
                            :*: ******  .  :*   : .*   . * :*..:. :   : ::        ::  : :
```

```
gi|133332|RPB1A_TRYBB        MLRADPQNVRKLQEEYEQLTADREWS-RKMLDLEDRDKLKLNLPVNPGRLIQNARSTMGK
3H0G:A|PDBID|S. Pombe        ----DSSVQDLLDEEYTQLVADRELLCKFIFPKGDA---RWPLPVNVQRIIQNALQIFHL
5FLM:A|PDBID|Bos Taurus      DVLSNAHIQNELEREFERMREDREVL-RVIFPTGDS---KVVLPCNLLRMIWNAQKIFHI
                                 :.      *: *: .:  ***    . ::    *     .  ** *  *:*.** . :

gi|133332|RPB1A_TRYBB        RSQV-SNLSPITIIDHVRKLQEDLMKLFPSYHRGGDGYIRNTLSRERIESALTLFNVHLR
3H0G:A|PDBID|S. Pombe        EAKKPTDLLPSDIINGLNELIAKLTIF-----RGSD-----RITRDVQNNATLLFQILLR
5FLM:A|PDBID|Bos Taurus      NPRLPSDLHPIKVVEGVKELSKKLVIV-----NGDD-----PLSRQAQENATLLFNIHLR
                              ..   ::* *  ::: :.:*  .*  .      .*.*       ::*:  :.*  **:: **

gi|133332|RPB1A_TRYBB        QLLASKRVLKEYKLNDRAFEYLLKEIRTKYHQSLTTPGENIGAIAAQSCGEPATQMTLNT
3H0G:A|PDBID|S. Pombe        SKFAVKRVIMEYRLNKVAFEWIMGEVEARFQQAVVSPGEMVGTLAAQSIGEPATQMTLNT
5FLM:A|PDBID|Bos Taurus      STLCSRRMAEEFRLSGEAFDWLLGEIESKFNQAIAHPGEMVGALAAQSLGEPATQMTLNT
                             . :. .*:   *:.*.  **::::: *: :.::*::. *** :*::**** ***********

gi|133332|RPB1A_TRYBB        FHNAGISSKNVTLGVPRLLELLNVSRNQKHASMTVSLFPPYDEKRNAQKAQHL---IEYC
3H0G:A|PDBID|S. Pombe        FHYAGVSSKNVTLGVPRLKEILNVAKNIKTPSLTIYLMP--WIAANMDLAKNVQTQIEHT
5FLM:A|PDBID|Bos Taurus      FHYAGVSAKNVTLGVPRLKELINISKKPKTPSLTVFLLG--QSARDAERAKDILCRLEHT
                             ** **:*:********** *::*::.: * .*:*: *:         : : *: :   :*:

gi|133332|RPB1A_TRYBB        TLESITRRIQFIYDPDPRHTVVEADRDILELEWNVMDESDAELRIQEVVAGSPWVVRLEL
3H0G:A|PDBID|S. Pombe        TLSTVTSATEIHYDPDPQDTVIEEDKDFVEAFFAIPDEEVEENLYKQ----SPWLLRLEL
5FLM:A|PDBID|Bos Taurus      TLRKVTANTAIYYDPNPQSTVVAEDQEWVNVYYEMPDFDVARI--------SPWLLRVEL
                             ** .:*     : ***:*. **:   *.: ::  : : * .             ***::*:**

gi|133332|RPB1A_TRYBB        DVDMVTDKALDMKDVKQAILRVDESYIIETGMANNVRQRTIRMR------SRYNEGADSI
3H0G:A|PDBID|S. Pombe        DRAKMLDKKLSMSDVAGKIAESFERDLFTIWSEDNADKLIIRCRIIRDDDRKAEDDDNMI
5FLM:A|PDBID|Bos Taurus      DRKHMTDRKLTMEQIAEKINAGFGDDLNCIFNDDNAEKLVLRIRIMNSDENKMQEEEEVV
                             *    : *. * *.::     *        :       :*. :  :* *        . ::  : :

gi|133332|RPB1A_TRYBB        PKLKREI------PALLARVHLRGIPGVRRALLK----DTTEFTVDQATGKMSGNKIWAI
3H0G:A|PDBID|S. Pombe        ---EEDVFLKTIEGHMLESISLRGVPNITRVYMM-----EHKIVRQIEDGTFERADEWVL
5FLM:A|PDBID|Mammalian       DKMDDDVFLRCIESNMLTDMTLQGIEQISKVYMHLPQTDNKKKIIITEDGEFKALQEWIL
                                . ::         :*  : *.*:  : .. :          :        * :.  . * :

gi|133332|RPB1A_TRYBB        DTDGTALRRAFIGVVGEDGKNIINAVKTSSNKVPEVCSLLGIEAARSKMLTELREAYLAY
3H0G:A|PDBID|S. Pombe        ETDGINLTE----AMTVEG---VDATRTYSNSFVEILQILGIEATRSALLKELRNVIEFD
5FLM:A|PDBID|Bos Taurus      ETDGVSLMR----VLSEKD---VDPVRTTSNDIVEIFTVLGIEAVRKALERELYHVISFD
                             :***   *       .:   ..    ::...* **.. *:   :*****.*. :   **    .

gi|133332|RPB1A_TRYBB        GLNINYRHYTILVDTICQHGYLMAVSRSGINRSDTSGPLMRCSFEETVKVLMAAASFGEC
3H0G:A|PDBID|S. Pombe        GSYVNYRHLALLCDVMTSRGHLMAITRHGINRAET-GALMRCSFEETVEILMDAAASGEK
5FLM:A|PDBID|Bos Taurus      GSYVNYRHLALLCDTMTCRGHLMAITRHGVNRQDT-GPLMKCSFEETVDVLMEAAAHGES
                             *   :**** ::* *.:   .*:***::* *:** :* *.**.*******.:** **: **

gi|133332|RPB1A_TRYBB        DPVRGVSANLVLGNQARVGTGLFDLVL
3H0G:A|PDBID|S. Pombe        DDCKGISENIMLGQLAPMGTGAFDIYL
5FLM:A|PDBID|Bos Taurus      DPMKGVSENIMLGQLAPAGTGCFDLLL
                             *   .*:* *::**: *   *** **: *
```
